# Milk-Sickness

**Published:** 1840-07

**Authors:** 


### MILK-SICKNESS.

When lately at Urbana, Ohio, we collected from Dr. Carter, who has practised medicine in that town for more than a quarter of a century, the following facts, relative to the production of "Milk-Sickness," or "Sick-Stomach." Formerly the disease prevailed much more than within the last 8 or 10 years. This, Doctor C. ascribes to the confinement of cows within enclosed pastures. The neighborhood of Urbana presents, at the surface, two geological varieties: first, a true upland level, clayey, and covered chiefly with white oak, the soil thin, and reposing on a compact, arenaceous, ash-grey limestone: second, a diluvial formation, consisting in extensive, level deposites of rolled pebbles, gravel and sand, covered with black, carbonaceous mould, destititute of trees and clothed in long grass. These tracts are evidently the beds of obsolete ponds and little lakes.

As long as cattle feed on the latter, according to the observation of Dr. Carter, the milk-sickness is not produced; when they frequent the woodlands, the disease is apt to occur. But it is not dangerous for them to feed in these natural pastures at *all* seasons of the year. Dr. Carter has never known them nor the people affected before the month of July, and, in most instances, not till August; from which till December is the time of greatest danger. He has never seen a case of the disease in man, that he could not trace up to the cow. Milch cows occasionally die, but on the whole are less liable than cattle which do not give milk. He knows of one tract of woodland three or four miles north-east of Urbana, which has been peculiarly fatal. Nearly all the following facts connect themselves with that spot.

Sixteen or eighteen years ago, a family living near it, had one milch cow, which was suffered to feed upon it. During the first August after their removal thither, and while they were using her milk, the father of the family sickened with the characteristic symptoms and died; and within a week from his death, the wife and two children, experienced the same fate, from attacks of the same kind

A young man living in the family had, at the same time, an attack of dysentery;—suggesting, undoubtedly, that the *gastric* disease which carried *them* off might have arisen from the same cause which produced *his intestinal* complaint. The cow was seized with the "*trembles,*" about the time the children were attacked, and died.

In the summer of 1837, in the month of August, the milch cows of Mr. Taylor, were suffered to range for a week in the same wood, during which they were milked as usual. The milk and butter made from it were eaten. A portion was also manufactured into cheese. Four of the family, two adults, and two children, all of whom had used the milk and butter, sickened with the well known symptoms, and one of the former died. The cheese was "thrown to the dogs"—one ate of it and sickened; and some chickens which picked up fragments of it died.

In the month of October 1839, about twenty cattle escaping from their enclosures near this wood, were allowed to graze, or rather to browse, in it for 6 or 7 days, when several of them were found dead on the ground. The remainder were driven home, affected with the *trembles.* Some of them died the first night, and but one of the whole recoveied.

Dr. Carter introduced us to Mr. Hitt, an intelligent and respectable farmer, who lives near Urbana, who informed us that he had lost many cattle by this malady; but has never seen it affect any which did not run at large, nor known it occur earlier in the calendar year than August. Two years ago in that month, his cattle ran abroad and one of the finest of them "got the trembles." He kept her from water, fed her on green corn (maize,) and drenched her with lard, as curative means. Finding that she did not improve in health, he determined to give her drink. After taking a few swallows of cold water, she fell down in convulsions, and to put an end to her sufferings he cut her throat. His two dogs ate of her carcase. In two days, one of them stiffened in his limbs "so that he could not jump over a low fence" and died—the other was affected with the same stiffening, but got well. His hogs, also, ate of the carcase, but were uninjured; but at another time, when he lost four cows, his hogs devoured a part of their carcases, and were destroyed.

We give these facts, with the observation that Dr. Carter is a regularly educated physician, and an honest man. D.

